# Optic Flow Processing in Patients With Macular Degeneration

**DOI:** 10.1167/iovs.63.12.21

**Published:** 2022-11-15

**Authors:** Jade Guénot, Yves Trotter, Paul Fricker, Marta Cherubini, Vincent Soler, Benoit R. Cottereau

**Affiliations:** 1 Centre de Recherche Cerveau et Cognition, Université Toulouse III–Paul Sabatier, Toulouse, France; 2 Centre National de la Recherche Scientifique, Toulouse Cedex–CNRS: UMR5549, Toulouse, France; 3 Unité de rétine, consultation d'ophtalmologie, hôpital Pierre-Paul-Riquet, CHU Toulouse, Toulouse, France

**Keywords:** optic flow, macular degeneration, AMD, Stargardt disease, motion processing, aging

## Abstract

**Purpose:**

Optic flow processing was characterized in patients with macular degeneration (MD).

**Methods:**

Twelve patients with dense bilateral scotomas and 12 age- and gender-matched control participants performed psychophysical experiments. Stimuli were dynamic random-dot kinematograms projected on a large screen. For each component of optic flow (translational, radial, and rotational), we estimated motion coherence discrimination thresholds in our participants using an adaptive Bayesian procedure.

**Results:**

Thresholds for translational, rotational, and radial patterns were comparable between patients and their matched control participants. A negative correlation was observed in patients between the time since MD diagnosis and coherence thresholds for translational patterns.

**Conclusions:**

Our results suggest that in patients with MD, selectivity to optic flow patterns is preserved.

Macular degeneration (MD) is the leading cause of legal blindness in countries with an aging population. A recent meta-analysis estimated that 67 million people are affected in Europe, and this number is expected to increase by 15% by 2050.^1^ Patients with MD experience a gradual loss of central vision that strongly impairs their ability to perform tasks such as reading^2^^,^^3^ or face recognition.^4^^,^^5^ The evolution of visual functions that mostly rely on peripheral vision has been much less explored in patients. In particular, very little is known about their ability to process motion and more particularly optic flow—the projection of the visual scene on the retinas during self-displacement.^6^ Optic flow can be decomposed into different visual patterns (translational and rotational, both due to eye and/or head rotation and radial due to observer forward/backward displacements) that are processed by distinct neural populations in the brain.^7^^,^^8^ This is crucial for heading perception,^9^^,^^10^ path integration,^11^ and estimating object movement during navigation (flow parsing).^12^ Its processing is even more important in aged populations, who more strongly rely on vision than on other senses during their displacements.^13^^,^^14^

Optic flow processing is believed to mostly rely on peripheral vision,^15^^,^^16^ although some studies supported the idea that central vision might also contribute.^17^ Peripheral stimulation can notably determine the sensation of vection.^18^ Optic flow processing could therefore be preserved in patients with MD as their peripheral vision remains functional. In line with this hypothesis, Tarita-Nistor et al.^19^ found that rotational and translational optic flow patterns elicited strong perception of motion in patients with MD. The correlation between their perceptual reports and objective measures of head tilt was actually more significant than that measured in a group of age-matched controls. Another study found that the sensation of vection elicited by a radial pattern was stronger in patients with MD than in controls with normal visual fields.^20^ Altogether, these two studies suggest that optic flow processing could even be enhanced in patients, possibly through functional reorganizations occurring after the onset of the scotoma and improving perception in peripheral vision. However, they only characterized the sensation of self-motion and did not investigate whether patients with MD could also outperform control participants in other visual tasks based on optic flow (e.g., in fine motion discrimination tasks).

One previous study^21^ measured motion direction and speed discrimination thresholds in patients with MD using wide-field translations (random-dot kinematograms moving along one direction) presented in peripheral vision. Thresholds did not significantly differ between patients with MD and their age-matched controls. Odom et al. (Odom JV, et al. *IOVS* 2010;51:ARVO E-Abstract 3619) asked their participants to determine the location of the focus of expansion (FoE) of a radial optic flow pattern and found that heading precision was impaired in patients with MD when stimuli were noisy. Finally, although they did not use full-field stimuli (and therefore focused more on local motion processing), Eisenbarth et al.^22^ found that patients had higher contrast thresholds than age-matched controls when discriminating the motion direction of a moving plaid presented at eccentricities up to 20°. Even though it is difficult to know exactly which part of the retina was stimulated in this case because the authors did not measure the preferred retinal locus (PRL) of patients (defined by Crossland et al.^23^ as a region of the functioning retina, repeatedly aligned with a visual target for a specific task, that may also be used for attentional deployment and as the oculomotor reference), this result nonetheless suggests that their processing of dynamic stimuli could be impaired beyond the border of the scotoma. Altogether, these studies support the idea that when considering fine discrimination tasks, performances of patients with MD are not enhanced as in the studies described above^19^^,^^20^ and could even be impaired, even though stimuli are presented in peripheral vision.

To date, no study has assessed how patients with MD process the three components of optic flow (translational, rotational, and radial). Here, we used a motion direction discrimination task combined with an adaptive Bayesian psychophysical procedure to robustly estimate motion coherence thresholds for each of these components in a group of patients with MD and in a group of gender- and age-matched controls. The experiments in the control group were performed using simulated scotomas of the same dimensions as those of their corresponding patients so that the two populations receive comparable visual inputs. Measurements in the control group were also reproduced without a simulated scotoma to evaluate the contribution of central vision in our task.

## Methods

### Participants

Thirteen patients were initially included in the study. One of them (87 years old) was not able to perform the task correctly and was excluded. We therefore collected the data from 12 patients (six females, mean age, 64.67 ± 14.72 years) with central vision loss. Six of them had a confirmed diagnosis of age-related MD (AMD), three of them with atrophic MD and three with Stargardt disease. The main clinical data of these patients (sex, age, time since MD diagnosis, diagnosis, visual acuity, tested eye, scotoma area, PRL eccentricity, and polar angle) are provided in the Table. The psychophysical tasks were performed monocularly. The patients were recruited from the Retina Center of the Purpan Hospital in Toulouse, France, where they underwent ophthalmologic examinations. Only patients who met all the inclusion criteria were included in the study.

**Table.** Summary of Characteristics of Patients and Age-Matched Controls Included in the Study

					Visual Acuity (logMAR)					
Subject	Sex	Age, y	Time Since MD Diagnosis, y	Diagnosis	OD	OS	Tested Eye	Scotoma Area, deg²	PRL Eccentricity, deg	PRL Polar Angle, deg
MD1	F	57	20	Stargardt	1.17	0.71	OS	15.36	10.58	265.93
MD2	M	63	8	Atrophic MD	0.66	0.56	OD	6.6	2.49	322.19
MD3	M	26	10	Stargardt	1.23	1.16	OD	12.96	14.97	329.19
MD4	F	58	23	Atrophic MD	0.76	≥1.72	OD	12.18	3.10	187.43
MD5	F	62	7	Atrophic MD	0.68	0.54	OS	14.94	8.94	189.73
MD6	M	59	8	Stargardt	0.63	0.5	OS	7.8	4.75	268.43
MD7	M	80	8	AMD	0.42	≥1.72	OD	8.52	2.21	302.25
MD8	F	71	9	AMD	0.8	0.75	OD	20	15.56	315
MD9	M	71	5	AMD	≥1.72	0.47	OS	20	4.17	342.56
MD10	F	74	6	AMD	0.95	0.88	OS	20	12.40	204.29
MD11	M	75	2	AMD	0.82	1.61	OD	7.72	3.99	101.56
MD12	F	80	5	AMD	0.49	0.55	OD	14.48	4.10	255.89
C1	F	56	—	—	–0.09	–0.1	OS	—	—	—
C2	M	62	—	—	–0.01	–0.16	OD	—	—	—
C3	M	27	—	—	–0.21	–0.17	OD	—	—	—
C4	F	61	—	—	0.02	–0.15	OD	—	—	—
C5	F	62	—	—	0.01	0.02	OS	—	—	—
C6	M	57	—	—	0.12	0.11	OS	—	—	—
C7	M	80	—	—	0.08	–0.05	OD	—	—	—
C8	F	71	—	—	0.15	0.25	OD	—	—	—
C9	M	71	—	—	0.14	–0.08	OS	—	—	—
C10	F	76	—	—	–0.01	0.09	OS	—	—	—
C11	M	75	—	—	0.11	0.38	OD	—	—	—
C12	F	76	—	—	0.15	0.23	OD	—	—	—

Scotoma area and PRL polar coordinates (eccentricity and angle) correspond to the tested eye. OD, right eye; OS, left eye.

Inclusion criteria were as follows: absolute central binocular scotoma of less than 20° in diameter, measured by 30° and 12° central visual fields analyzed with the Octopus 300 perimeter (Haag-Streit, Köniz, Switzerland) and with spectral-domain optical coherence tomography (Spectralis Optical Coherence Tomography (OCT); Heidelberg Engineering, Heidelberg, Germany; see the “Scotoma Size” section); age between 18 and 90 years; residual vision in both eyes (i.e., visual acuity between 1.3 and 0.5 logMAR); and stable ocular fixation (i.e., presence of a single PRL as detected with OCT measurements; see the “PRL Localization” section).

Patients with a concomitant presence of other visual diseases (cataract, retinal detachment, glaucoma), an inability to understand the task, or unilateral MD were excluded.

The control group consisted of 12 age- and gender-matched participants (six females; mean age, 64.5 ± 14.33 years; see the Table). All the control participants had a corrected visual acuity over 6/10 on the tested eye. They were recruited via advertisements in local journals and on social media.

Both patients and controls gave written informed consent. The research was conducted at the Centre de Recherche et Cognition (Toulouse, France), and the experimental protocol was approved by a national ethics committee before the beginning of the study (CPP, Comité de Protection des Personnes, protocols 13018–14/04/2014 and 2020-A02441-38).

### Scotoma Size

**Figure 1.**
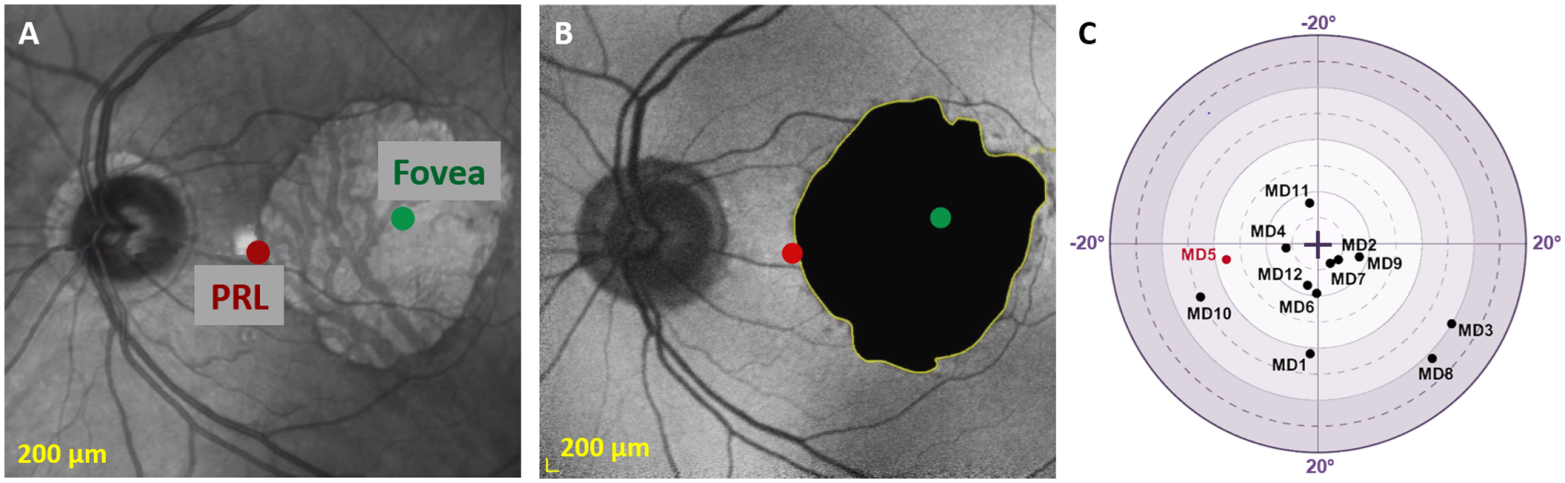
Standard (**A**) and autofluorescence (**B**) imaging of the eye fundus of one typical patient (MD5). The macular atrophy (circled in *yellow* in **B**) was delineated by an ophthalmologist. The PRL and fovea of this patient are respectively given by the *red* and *green dots*. Note that the eye fundus in these two panels has been flipped relative to the horizontal meridian to be aligned with the visual field. (**C**) Positions of the PRLs for the 12 patients with MD. The PRL of patient MD5 (used in **A** and **B**) is marked in *red*.

For each patient, an ophthalmologist delineated the border of the scotoma from the fundus of the eye captured by standard and autofluorescence imaging (see Fig. 1).

The area of the scotoma (in deg² of visual angles) was extracted using MATLAB (MathWorks, Natick, MA, USA). This area was used to compute the simulated scotoma of the age- and gender-matched controls (see more details in “Experimental Design” section).

### PRL Localization

For each patient with MD and for each eye, we estimated the position of the PRL with respect to the fovea (see Fig. 1) from high-resolution scans of the retinal fundus acquired with spectral-domain optical coherence tomography (Spectralis OCT; Heidelberg Engineering) using a method previously described by Maniglia et al.^24^ (see Supplementary Text 1 for a detailed explanation). Measures of the PRL coordinates were repeated three times to make sure that patients had a stable ocular fixation (i.e., less than 2° of variability across measurements, based on the ocular fixation classification proposed by previous studies).^25^^,^^26^ The final PRL coordinates were obtained by averaging these three measurements together (see the Table) and were subsequently converted into polar coordinates.

### Determination of the Tested Eye

The visual acuity of each participant was determined for each eye using the Sloan letters of the Freiburg Visual Acuity Test.^27^ In the subsequent psychophysical tasks, each patient was tested monocularly in the eye with the better acuity (the other eye was patched) except for MD2 and MD3, whose ocular fixation (see the previous paragraph) was much more stable with the other eye, and for MD8, who had a macular hemorrhage on her best eye when she performed the study. Age-matched controls were tested on the same eye as their matched patient.

### Motion Stimuli

**Figure 2.**
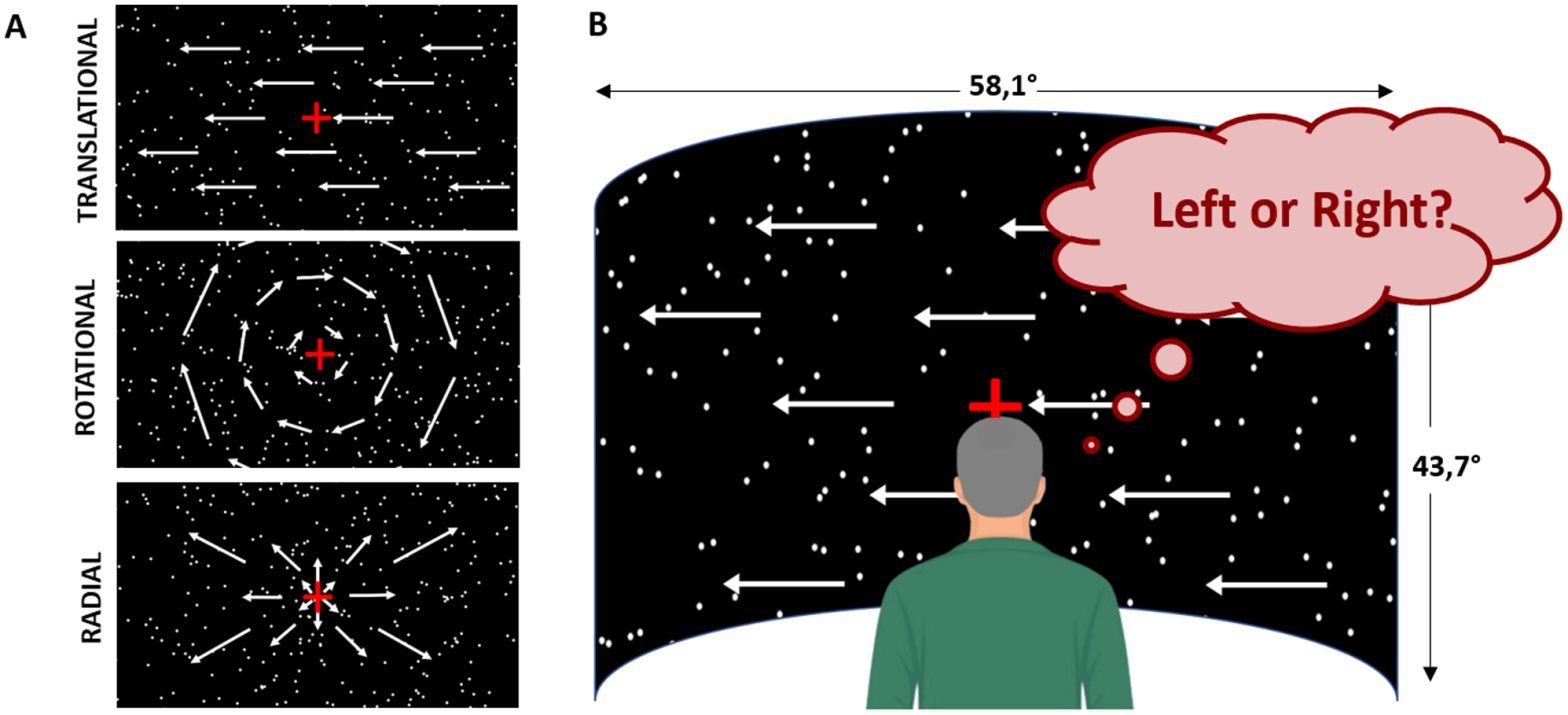
Stimuli and experimental protocol. (**A**) Stimuli were random-dot kinematograms (RDKs) moving in different directions. We used three components of optic flow: translational (*upper*), rotational (*central*), and radial (*lower panel*). (**B**) RDKs were projected on a large convex screen at a viewing distance of 180 cm. Participants were instructed to fixate on a red cross and to report the motion direction of the stimuli (leftward versus rightward for translational motion, clockwise versus counterclockwise for rotational motion, and inward versus outward for radial motion). We manipulated motion coherence parametrically and estimated the thresholds corresponding to 80% of correct detection.

Visual stimuli were created in MATLAB (R2017a) using the Psychophysics toolbox^28^^,^^29^ and consisted of random-dot kinematograms projected on a large convex screen (58.1° × 43.7° of visual angle, refresh rate: 60 Hz, resolution: 1400 × 1050 pixels) in a dark room and at a viewing distance of 180 cm. Dots were bright disks (0.2° in diameter moving on a dark background, contrast: 100%). They had a density of 0.3945 dots per degree of visual angle. We adapted the protocol described in a previous electrophysiologic study^30^ to define motion patterns. Each dot had a lifetime of 200 ms (12 frames), during which it moved along a straight line at a constant speed. At the end of this lifetime, it was randomly reassigned to a new location within the screen and given a trajectory and speed corresponding to this new location. To avoid a coherent flickering of the stimulus every 200 ms, each dot initial age was randomly picked between 0 and 166 ms (11 frames) at the beginning of each trial. Dots reaching the border of the screen were immediately relocated at a new position. These processes permit equalizing dot density and mean luminance across the screen during the experiment. We used three different types of optic flow patterns: translational, radial, and rotational (see Fig. 2A). Speed in the translation condition was equal to 7°/s, a value that corresponds to the average preferred speed of MT neurons measured in macaques.^31^ The radial and rotational optic flow patterns had identical speed distributions. In these conditions, the speed of a dot was a function of its eccentricity (Ecc) and was given by S × Ecc. S was chosen to equalize the average speed in the radial and rotational conditions with the speed in the translational condition, in order to obtain comparable thresholds between the three optic flow components. Because the trajectories contained no curvature or acceleration and because dot size, density, and speed were equalized between the translational, radial, and rotational optic flow patterns, the only difference between these three conditions was the global motion.

### Experimental Design

During the experiments, participants sat in a chair whose height was adapted so that their eyes were at the same level as the center of the screen. Their head was placed on a head-support device clamped on top of a table and equipped with both chin and forehead supports. The chair and head-support devices were positioned so as to ensure a fine alignment between the participants’ head and trunk axes. Participants were instructed to keep this position as constant as possible.

Stimuli were presented in blocks of 64 trials and about 3 minutes using the Psychophysics toolbox.^28^ Only one optic flow pattern (translation, rotation, or expansion/contraction) was used during a block. Participants were involved in a two-alternative forced-choice task. For each trial, they had to report the motion direction of the stimuli (leftward versus rightward for translational, clockwise versus anticlockwise for rotational patterns, and inward versus outward for radial). Stimuli were presented for 200 ms and participants had up to 2 seconds to respond. Responses reported after this time limit were considered incorrect. Importantly, given the short duration and relatively low speed of our stimuli (see above), it is unlikely that they elicited vection (as a comparison, Tarita-Nistor et al.^19^ used translational stimuli moving at 60°/s for 2 minutes in their study on vection). Our participants therefore reported the motion direction of the stimuli rather than their self-motion perception. During each block, we manipulated motion coherency (i.e., the percentage of dots moving in the same direction while the other dots had random directions) and estimated the thresholds corresponding to 80% of correct detection using a Bayesian adaptive psychometric method (QUEST, see below). The three first blocks (one for each optic flow pattern) were considered a training and were thus discarded from further analyses.

**Figure 3.**
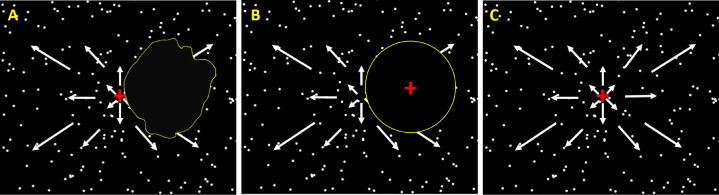
Schematic representation of visual inputs during the task. (**A**) Patients with MD gazed at a central fixation red cross with their PRL. Their scotoma (circled in *yellow*) masked one portion of the visual field. (**B**) In the main viewing condition, controls gazed at a red fixation point presented at the position of the fovea of their paired patient, and their central vision was masked with a simulated scotoma. The size and the position of this simulated scotoma corresponded to the one measured in their paired patient. Illustrations in **A** and **B** are based on the clinical data (PRL and scotoma) of MD5 (as in Fig. 1). (**C**) In the second viewing condition, control participants gazed centrally and their visual field was unmasked.

During the psychophysical measurements, patients with MD were instructed to gaze monocularly on a central fixation cross with their PRL (see Fig. 3A). This central position was also the FoE of optic flow and coincided with the simulated heading for radial patterns. Control participants performed the experiments in two viewing conditions. In the main one, they had to gaze on a fixation cross presented at the position of the fovea of their paired patient, and their central vision was masked with a simulated scotoma, as in previous studies^32^^,^^33^ (see Fig. 3B). The size of this simulated scotoma corresponded to the one measured in their paired patient (see the “Scotoma Size” section). In this case, the center of the screen (and therefore the FoE for radial patterns) was projected on their peripheral vision, at the PRL location of their paired patient. This protocol permits patients and controls to receive comparable visual inputs (it notably equalizes the position and surface of the occluded part of the visual field) and thereby provides a very good framework to characterize the effect of macular degeneration on visual processing under wide-field stimulation. Because in this case, control participants did not gaze straight ahead, we measured their eye position with an eye-tracker to control that our measures were not affected by fixation instability. These analyses demonstrated that eye fixation was actually very stable in this case and similar to that measured under straight-ahead fixation. They are presented in supplementary materials (see Supplementary Text 2 and Supplementary Fig. S1). We did not measure eye position in patients with MD because calibration is very time-consuming in this population and would have significantly reduced the duration and hence the robustness of our psychophysical measures. We nonetheless controlled that they had a stable fixation with their PRL (see the “PRL Localization” section). In the second viewing condition, control participants gazed centrally and stimuli were presented without a simulated scotoma (see Fig. 3C). We used this control condition to determine the contribution of central vision in our task.

We ran three blocks for each optic flow pattern (translation, rotation, or expansion/contraction) in both patients with MD and controls. We included breaks (3 to 4 minutes on average) between blocks to reduce fatigue. Blocks and condition sequences were randomized to minimize possible learning effects on the results. The whole experiment was completed in one session of about an hour in patients. Two sessions of about the same duration were necessary in controls because measurements in this group included two viewing conditions (see above).

### Robust Estimation of the Motion Coherence Thresholds Using a Bayesian Adaptive Psychometric Method

Because it is difficult to perform long psychophysical measurements in aged populations, we estimated motion coherence thresholds using the QUEST adaptive procedure, which permits rapid and robust estimations. QUEST is a Bayesian method that assumes that the psychophysics function underlying the performance of the participants follows a Weibull distribution. During a block, the estimated parameters of this function were updated after each trial on the basis of the participant's performance. Coherence values corresponded to the current maximum likelihood estimate of the threshold. We fixed the maximum number of trials at 64 as it was previously shown that this value leads to robust thresholds in most circumstances.^34^ We used an initial threshold value of 58% based on pilot recordings performed in patients and age-matched controls. The robustness and reproducibility of our psychophysical protocol were also validated from tests–retests performed in two patients and four controls. Results showed that the estimated thresholds were very stable across both blocks and sessions.

### Statistical Analysis

For each participant and each optic flow pattern (translational, rotational, and radial), we averaged together the thresholds estimated in the three recording blocks. To compare the performances of the control participants with and without a simulated scotoma, we used a two-way ANOVA with the optic flow pattern (translational, rotational, or radial) and the viewing condition (with or without an artificial scotoma) as within-subject factors. To compare performances between the groups of patients and controls, we used a two-way ANOVA with the optic flow patterns (translational, rotational, or radial) as within-subject factor and the group (patient or control) as the between-subject factor. Sphericity was always respected, and thus, we did not apply any corrections. Significant effects of the ANOVA were explored with post hoc paired *t*-tests, corrected for multiple comparisons (Bonferroni). The strength of the relationships between the measured thresholds and age, visual acuity, scotoma size, and time since MD diagnosis was assessed with Pearson *r* correlations.

## Results

The aim of this study was to characterize the impact of macular degeneration on optic flow processing. We estimated psychophysical thresholds during a motion discrimination task performed by patients and age-matched control participants with and without a simulated scotoma.

### Impact of Central Vision on Optic Flow Processing for Control Participants

**Figure 4.**
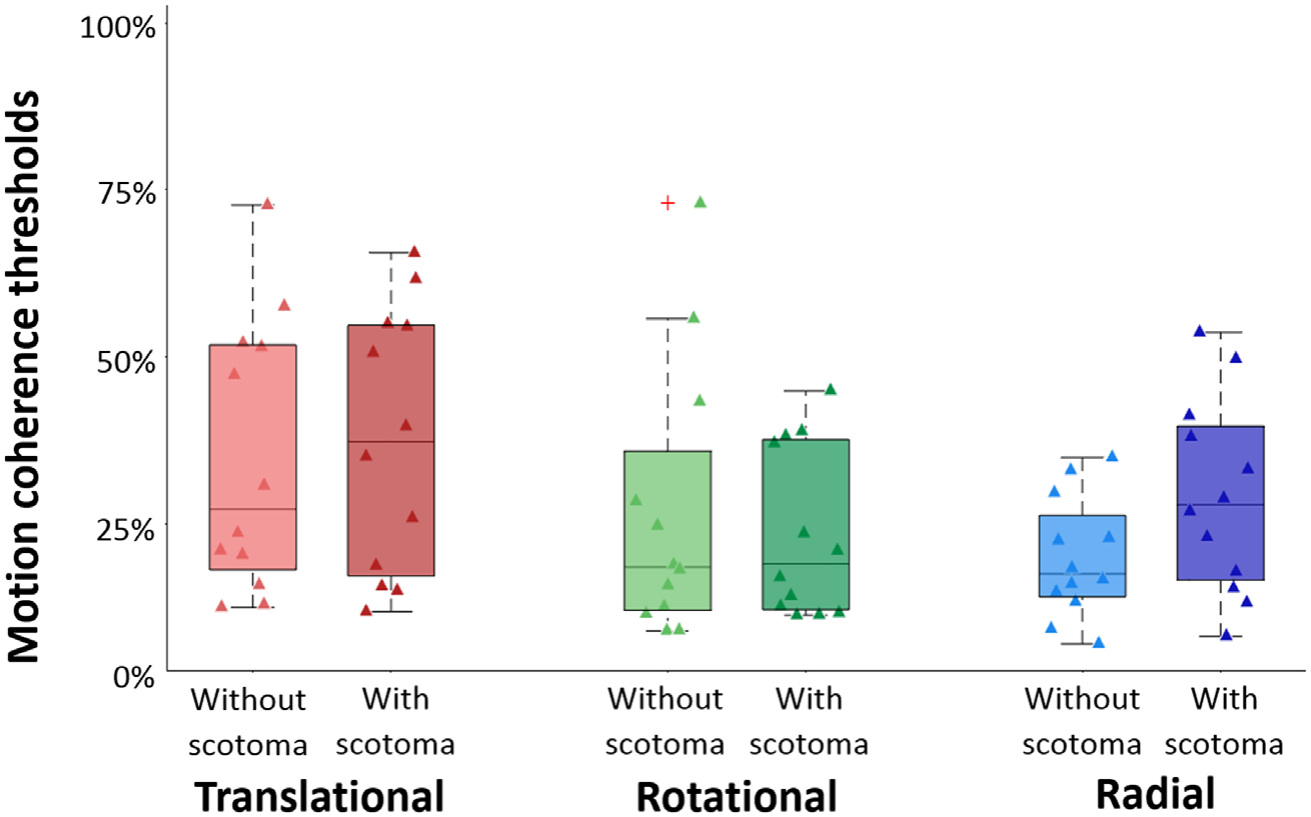
Motion coherence thresholds estimated for translational (*red*), rotational (*green*), and radial (*blue*) patterns. The *boxplots* show the different values for the first quartile, median, last quartile, and the extreme data points. *Light* and *dark boxes* respectively represent data from age-matched controls in the conditions without scotoma (*light colors*) and with scotoma (*dark colors*). *Triangles* provide the individual data points of the distributions. Note that they were slightly offset horizontally to improve their visibility.

Before analyzing data in patients, we determined the influence of central vision on optic flow processing in our control participants. Figure 4 shows the distributions of thresholds measured in this group with and without a simulated scotoma for translational (in red), rotational (in green), and radial (in blue) patterns.

In this case, statistical analyses consisted of a two-way ANOVA with the viewing condition (with or without a simulated scotoma) and the optic flow pattern (translational, rotational, or radial) as within-subject factors. This ANOVA did not lead to a significant effect of the viewing condition, *F*(1, 11) = 2.25, *P* = 0.162, or to a significant interaction between the viewing condition and the optic flow pattern, *F*(2, 22) = 1.44, *P* = 0.258 (see Fig. 4). It suggests that in control participants, optic flow processing is performed by peripheral vision. The ANOVA led to a significant effect of the optic flow pattern, *F*(2, 22) = 6.87, *P* = 0.004, driven by higher thresholds for translational (mean ± SD, 36.1 ± 19.67) than for rotational (25.03 ± 16.72, *P* = 0.036, for the paired post hoc *t*-test with a Bonferroni correction for multiple comparisons) and radial (24.43 ± 12.72, *P* = 0.015) patterns. Coherence thresholds did not significantly differ between rotational and radial patterns (*P* = 1.0).

### Comparison Between Motion Coherence Thresholds in Patients and in Age-Matched Controls With a Simulated Scotoma

We compared performances in patients and controls. Because we did not observe significant differences between the thresholds measured in the control group for the two viewing conditions (with and without a simulated scotoma; see above), here, we used only the data collected with a simulated scotoma.

**Figure 5.**
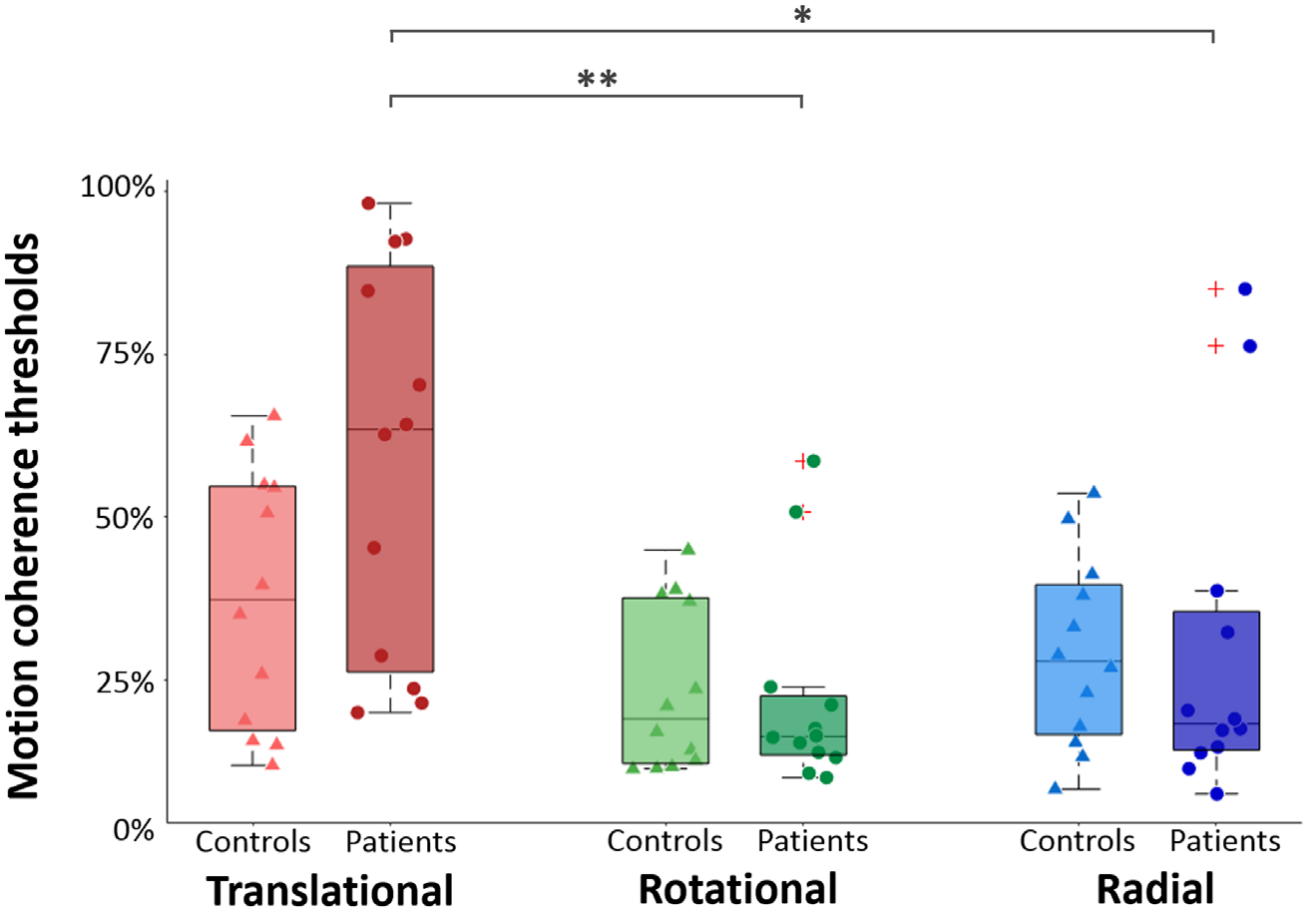
Motion coherence thresholds estimated for translational (*red*), rotational (*green*), and radial (*blue*) patterns. The *boxplots* show the different values for the first quartile, median, last quartile, and the extreme data points. *Dark* and *light boxes* respectively represent data from patients with MD and from their age-matched controls. Individual datapoints are respectively given by *triangles* (for controls) and *circles* (for patients with MD). Note that they were slightly offset horizontally to improve their visibility. *Stars* provide significant differences in the post hoc paired *t*-tests after a Bonferroni correction for multiple comparisons (see details in the text). *P < 0.05. **P < 0.01.

Figure 5 shows the distributions of these thresholds for translational (in red), rotational (in green), and radial (in blue) patterns. Statistical analyses consisted of a two-way ANOVA with the optic flow pattern (translational, rotational, or radial) as the within-subject factor and the group (patient or control) as the between-subject factor.

In agreement with the results observed in the previous section, this ANOVA led to a significant effect of the optic flow pattern, *F*(2, 44) = 14.29, *P* < 0.001. Here as well, post hoc *t*-tests indicated that motion coherence thresholds for the translational pattern (mean ± SD, 48.05 ± 27.14) were significantly higher than those for the rotational (22.84 ± 14.03, *P* < 0.001) and radial (29.23 ± 20.3, *P* = 0.009) patterns. No differences were observed between rotational and radial patterns (*P* = 0.897). The ANOVA did not lead to a group effect, *F*(1, 22) = 1.19, *P* = 0.287, but revealed a significant interaction between groups and patterns, *F*(2, 44) = 3.28, *P* = 0.047, although this effect was statistically smaller than the pattern effect. Post hoc pairwise *t*-tests corrected for multiple comparisons (Bonferroni) indicated that there were no significant differences between thresholds measured in the two groups for translational (*P* = 0.21), rotational (*P* = 0.887), and radial (*P* = 0.964) patterns. Paired *t*-tests also revealed that threshold differences between translational patterns, on the one hand, and the rotational and radial patterns, on the other hand, were significant in patients but not in controls (patients: respectively, *P* = 0.001 and *P* = 0.014; mean ± SD: translational, 58.7 ± 30.05; rotational, 22.24 ± 15.76; radial, 29.43 ± 25.5; controls: respectively, *P* = 1.0 and *P* = 1.0; mean ± SD: translational, 37.39 ± 20.39; rotational, 23.44 ± 12.74; radial, 29.04 ± 14.54).

In order to control whether the extreme data points measured in our experiment (i.e., the thresholds measured in two patients for rotational and radial optic flow patterns in Fig. 5) had an impact on our results, we reproduced the statistical analyses described above without these patients (and their associated controls) and found the same effects.

Altogether, these results suggest that in patients with MD, the processing of rotational, radial, and translational patterns of optic flow is preserved. Their robustness was controlled by reproducing the analyses using the threshold measured in control participants without a simulated scotoma. Effects remain unchanged in this case (see Supplementary Text 3 for more details).

### Correlations Between Motion Coherence Thresholds Estimated in Patients With MD and Clinical Data

Patients with MD are very different from one another, and there was a wide variety of clinical characteristics in our group of participants (i.e., scotoma size, time since the MD diagnosis, age, visual acuity, sex, etc.; see the Table). To test whether the motion coherence thresholds estimated for the three optic flow patterns were related to these data, we computed Pearson correlation coefficients.

**Figure 6.**
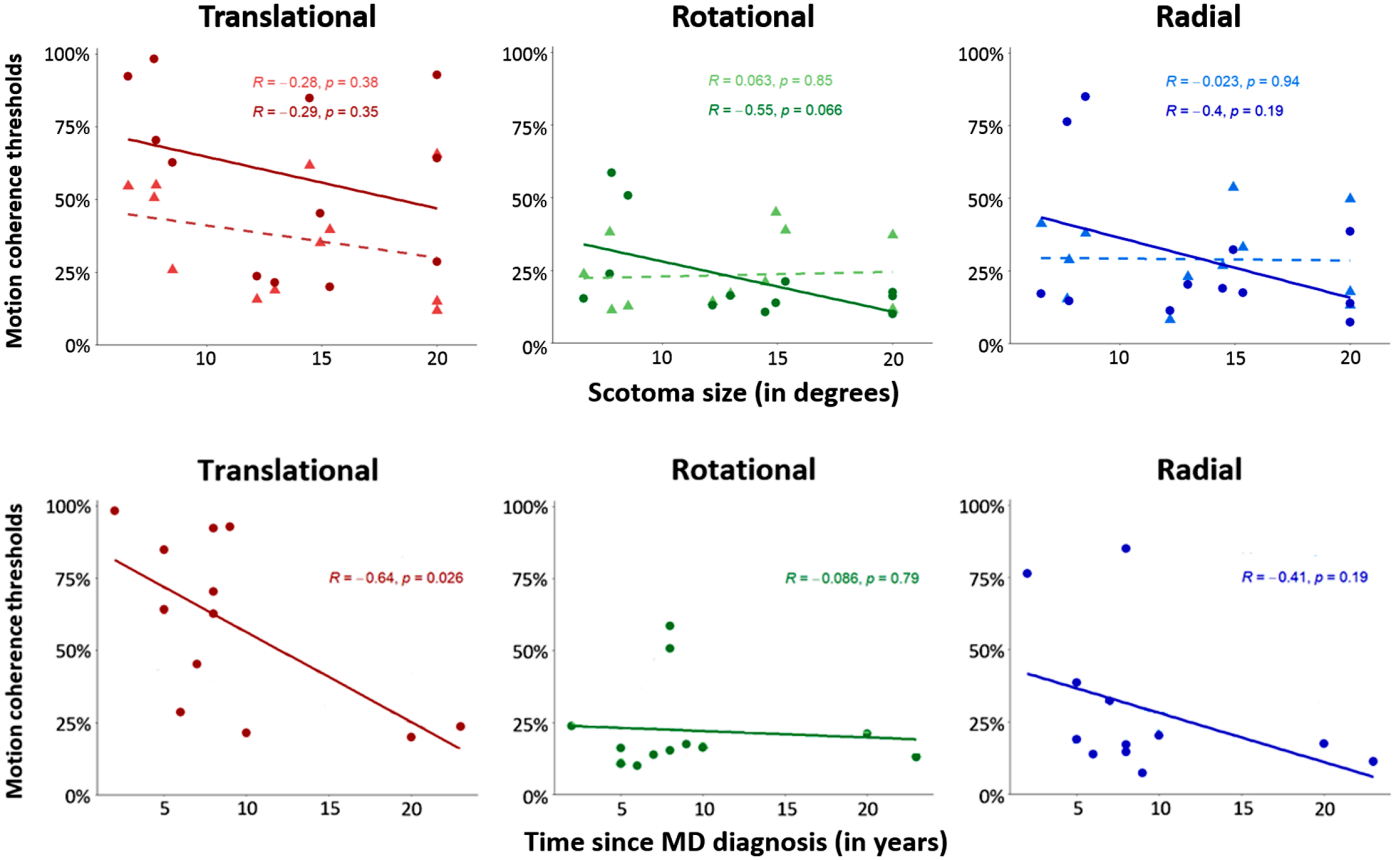
(**A**) Correlations between motion coherence thresholds and scotoma sizes (in degrees), estimated for translational (*red*, *leftward*), rotational (*green*, *middle*), and radial (*blue*, *rightward*) patterns. Data points and associated regression lines are provided for patients (*dark colors*) and also for their age-matched controls (*light colors*). Pearson correlation coefficients (*R*) and associated *P* values are provided on the *upper* parts of each panel. (**B**) Correlations between motion coherence thresholds and durations (in years) since the MD diagnosis.

The correlations between motion coherence thresholds and the two main clinical characteristics (i.e., scotoma size and time since the MD diagnosis) are presented in Figure 6. Other correlations are provided in Supplementary Figure S3. Scotoma size did not correlate with motion coherence thresholds measured for the three optic flow patterns (*P* > 0.05; see Fig. 6A). This result is in agreement with the idea that optic flow is mostly processed by peripheral vision in our experiment (see above). However, in patients, a significant negative correlation was found between time since the MD diagnosis and coherence thresholds for translational patterns, *r*(10) = –0.64, *P* = 0.026 (see Fig. 6B). No correlations were found between visual acuity and motion coherence thresholds (*P* > 0.05; see Supplementary Fig. S3B) or between age and motion coherence thresholds measured for the three optic flow patterns (*P* > 0.05; see Supplementary Fig. S3A) in controls or patients. However, in patients, a trend was observed for the correlation between age and threshold for translations, *r*(10) = 0.56, *P* = 0.058. This trend should be taken with care as the distribution of ages was skewed (one patient and his control were much younger than the other participants) but could indicate an effect of age on translation perception, in agreement with a previous study.^35^ This effect was not observed in the control group, *r*(10) = 0.27, *P* = 0.369.

Finally, for each optic flow pattern, we examined whether behavioral responses were biased toward one direction or the other (e.g., toward leftward or rightward motions for translational patterns). Our analyses of the correct responses did not lead to any significant bias in both patients and controls (see Supplementary Fig. S4). For translational patterns, we reproduced these analyses by taking into account the tested eye (to check for a bias toward temporal or nasal motions) and did not observe significant effects (see Supplementary Fig. S4D). We also did not observe differences between the motion coherence threshold measured in men and women (see Supplementary Fig. S5).

## Discussion

The aim of this study was to characterize the impact of MD and notably AMD on optic flow processing. This is important because the elderly mostly rely on visual cues during locomotion.^36^^,^^37^ We used an adaptive psychophysical approach to estimate motion coherence thresholds for translational, rotational, and radial patterns in patients with MD and in a group of gender- and age-matched controls with a simulated scotoma. Altogether, we found that despite their disease and notably their very low visual acuity, patients with central vision loss are still able to discriminate the motion direction of optic flow patterns.

Motion coherence thresholds for radial and rotational patterns had never been estimated in patients with MD. We found that they did not significantly differ between our two groups, which suggests that motion direction perception is preserved for these patterns in patients. One previous study based on preliminary data found that heading judgments could be impaired in patients when noise is added to radial stimuli (Odom JV, et al. *IOVS* 2010;51:ARVO E-Abstract 3619). Patients might therefore be affected for some visual functions (heading) but not for others (motion direction discrimination), and further explorations will be needed to get a full picture of the consequences of MD on the processing of radial and rotational optic flow patterns. Our results also suggest that thresholds for translational patterns are preserved in patients. In a previous study,^21^ patients with MD and age-matched controls performed motion direction and speed discrimination tasks with translational patterns presented in peripheral vision. In agreement with our measurements, thresholds did not significantly differ between the two groups. In another study based on local rather than full-field stimuli, Eisenbarth et al.^22^ reported that contrast thresholds were significantly higher in patients with MD than in a group of age-matched controls when discriminating the motion direction of a plaid presented at 10° and 20° of eccentricity. Our results are not in direct agreement with this study but used a different task based on local motion, which suggests that impairment in local motion processing does not necessarily imply a deficit in optic flow processing. Recordings from a larger population may clarify whether or not patients might be impaired in this case.

In a previous study performed by Tarita-Nistor et al.,^19^ patients with bilateral AMD and age-matched controls were exposed to translational and rotational optic flow patterns and had to report their sensation of self-motion (vection). Results showed that patients experienced vection sooner than controls, which suggests that self-induced motion might be improved in this population. In line with this study, Luu et al.^20^ reported higher vection strength and enhanced spatial presence in patients with AMD compared to healthy subjects when exposed to radial optic flow patterns. In our case, participants were instructed to report the motion direction of different optic flow patterns. We measured the associated coherence thresholds and not vection. Moreover, it is unlikely that our visual stimuli induced self-motion perception because of their short duration (200 ms) and relatively slow speed (7°/s on average). Our results therefore cannot be directly compared to those of Tarita-Nistor et al.^19^ and Luu et al.^20^ The fact that vection is enhanced in patients with central vision loss^19^^,^^20^ while motion perception is not indicates that the cortical networks underlying these two cognitive functions might substantially differ.

We found a significant negative correlation between motion coherence thresholds for translational patterns and time since the MD diagnosis in our group of patients (no other significant correlations were found between thresholds and data in the two groups). If this correlation should be interpreted with care and does not imply causality, it raises the interesting possibility that despite their disease and their age, patients with central visual loss might partially improve their ability to perceive translational motion over time, potentially through neural plasticity, as observed in animal models (e.g., Burnat et al.^38^ showed reorganizations of area MT/V5 and improved motion perception in adult cats a few months after a central retinal lesion). This idea opens promising perspectives for the development of future and innovative therapeutic approaches based on perceptual learning^24^^,^^39^^,^^40^ or oculomotor training.^41^

Beyond the comparison between performances in patients with MD and in controls, one of the main results of our study is that thresholds for translational patterns were significantly higher than thresholds for rotational and radial patterns in both groups. This effect is unlikely to be caused by differences in our stimuli because all three patterns were based on identical parameters (dot density, average speed, luminance, etc.; see the Methods section). One possibility is that it reflects an effect of age, as suggested by Billino et al.^35^ or Kuba et al.,^42^ who showed that aging impairs the perception of translational but not radial patterns. In our study, we had only two young participants (one 26-year-old patient and his age-matched control), and it is thus difficult to conclude on this point. Future studies should explore the validity of this hypothesis through psychophysical measurements in a large population of young and old participants.

Another important result of our study is that in the control group, motion coherence thresholds remained unchanged for visual stimulations with and without a simulated scotoma defined from clinical measurements in the paired patients. We used these simulated scotomas because previous studies reported that central vision might contribute to optic flow processing^17^ and because we wanted to make the signals received by each patient and his or her control as comparable as possible. It appears that under our experimental protocol, only peripheral vision was useful to perform the task, in line with the results of previous studies^15^^,^^16^ and with the idea that optic flow is processed within high-level visual areas whose neurons have large receptive fields.^43^^,^^44^

In our study, participants were instructed to gaze on a cross and not to move their head because it permitted to better control their retinal projections. During natural locomotion, the eyes and the head can move, which greatly complexify retinal projections. We know much less about optic flow processing in this more realistic case. Interestingly, in a recent study, Matthis et al.^45^ explored retinal optic flow in participants walking in real-world environments and found that their oculomotor strategy actually stabilized projections on their retina. It would be very interesting for future studies to test whether such mechanisms still exist in aged populations, including in patients with macular degeneration.

In conclusion, our results suggest that despite their low visual acuity and central vision loss, patients with macular degeneration are not impaired in their ability to perceive the direction of optic flow stimuli.

## Supplementary Material

Supplement 1
