# Supplementary material for: Optic Flow Processing in Patients With Macular Degeneration
Source: Invest Ophthalmol Vis Sci. 2022 Nov 15;63(12):21. doi: 10.1167/iovs.63.12.21 (PMC9672899; doi:10.1167/iovs.63.12.21)
Supplement: Supplement 1 [file iovs-63-12-21_s001.pdf]

Supplementary materials

Supplementary text 1: PRL Localization

For each patient with MD and for each eye, we estimated the position of the PRL with respect to the fovea using a method previously described in Maniglia et al.[23]. First, we localized the fovea on high-resolution scans of the retinal fundus centered on the atrophic macular area and acquired with spectral domain Optical Coherence Tomography (Spectralis OCT, Heildelberg Enginering, Heildelberg, Germany). Three additional landmarks (i.e., crossing blood vessels) were determined on these images and their coordinates were measured in microns with respect to the fovea. In a second step, patients were instructed to gaze with their PRL on a light point displayed on the center of the OCT screen while X-line OCT B-scans were acquired. We determined the positions of the three landmarks on these new scans which were centered on the PRL. The coordinates of the PRL (in microns) with respect to the fovea were finally determined using trilateration. Coordinates in microns were finally converted into coordinates in degrees of visual angle assuming that 288 μm on the retina corresponds to 1° of visual angle[46]. This second step was repeated three times to get three different values of the PRL coordinates. Between each measurement, patients closed their eyes and the OCT was defocused. This operation permitted to make sure that patients had a stable ocular fixation (i.e., less than 2° of variation across measurements, adapted from the fixation classification used by Fujii et al.[24] or Crossland et al.[25]). The final PRL coordinates were obtained by averaging the three measurements together (see table 1).

Supplementary text 2: Ocular fixation stability

Eye movements were recorded monocularly in the control group using an eye-tracker (EyeLink 1000, sampling frequency: 1kHz) placed at 35 cm in front of the participants. We did not

perform these recordings in patients because calibration in this population is often long and tiring and would have limited the time duration dedicated to our psychophysical measures. Data from control C6 were removed because this participant had glasses and his eye signal was extremely noisy. For each of the remaining control participants and each condition, we estimated the average eye position during the 200ms presentation of the stimuli. Trials with blinks were removed from the analysis to avoid artifacts. Figure S1-A provides a heatmap of the normalized fixation duration of control C5 (i.e., the control of MD5 which data were used for figures 1 and 3) during optic flow stimulation with a simulated scotoma (i.e., when gaze was directed on the fovea position of the paired patient). Although the fixation cross was eccentric in this case, we can observe that her gaze was accurate and very stable during the experiments. This result is also true at the group level as average eye positions relative to fixation in this viewing condition were also accurate and very stable for the three optic flow patterns (see figure S1-B). These average eye positions were actually similar to those measured in the viewing condition without a simulated scotoma (i.e., with a straight-ahead gaze, see figure S1-C).

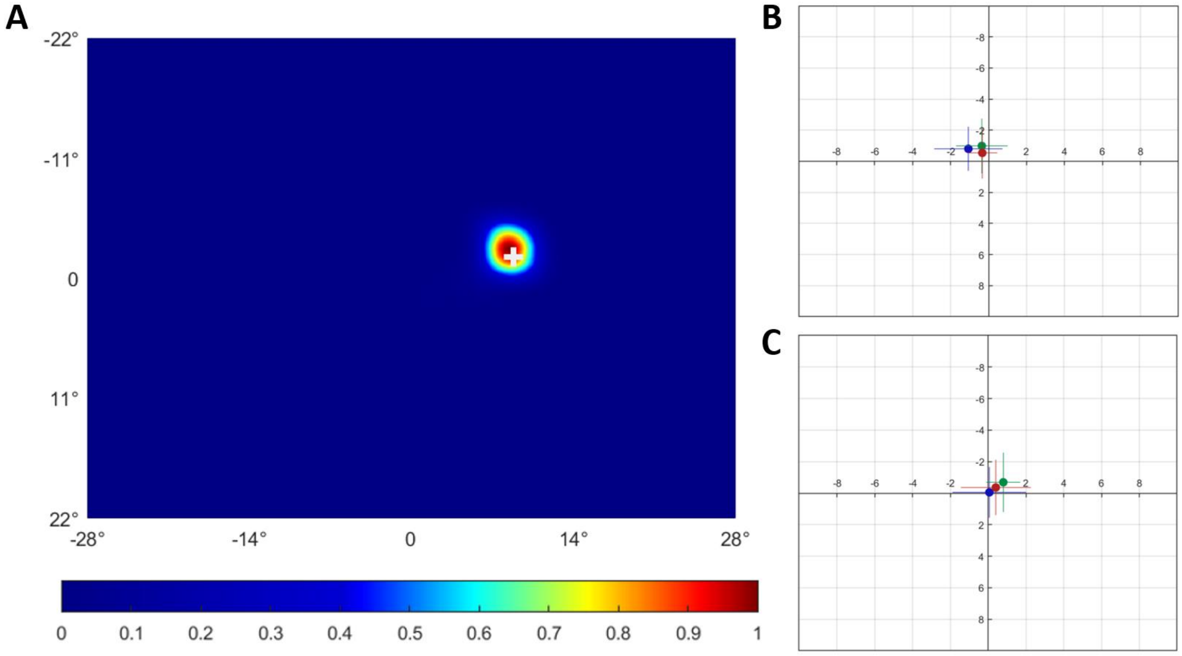

Figure S1. *(A) Normalized fixation duration in the age and gender-matched control of MD5 for the viewing condition with a matched scotoma. The point of ocular fixation is given by the white cross. (B)*

Average eye positions relative to the fixation cross in the control group for the viewing condition with a matched scotoma. The red, green and blue discs respectively represent these positions for translational, rotational and radial patterns. The horizontal and vertical segments provide the 95% confidence intervals on the x and y axes. (C) idem for the viewing condition without a matched scotoma (i.e., when ocular fixation was straight-ahead).

Supplementary text 3: Comparison between motion coherence thresholds in patients and in age-matched controls under normal viewing conditions

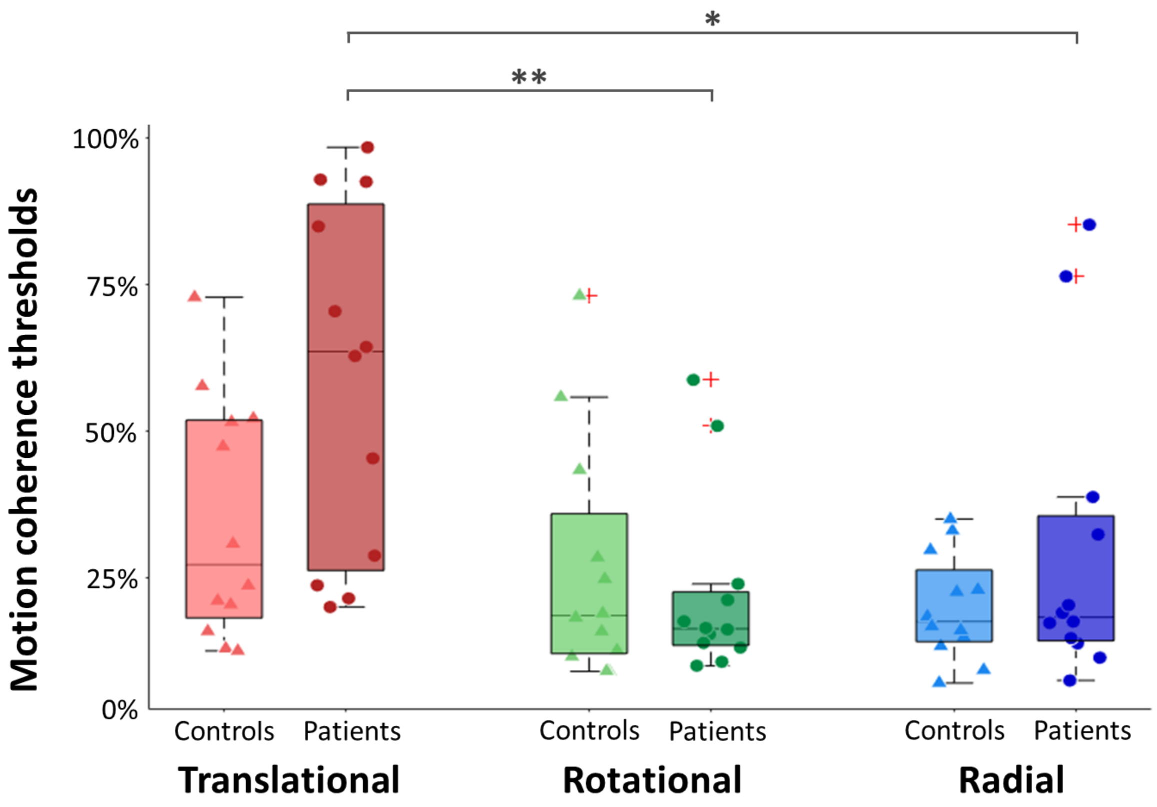

Figure S2. *Motion coherence thresholds estimated for translational (in red), rotational (in green) and radial (in blue) patterns. The boxplots show the different values for the first quartile, median, last quartile, and the extreme data points. Light and dark boxes respectively represent data from age-matched controls in the conditions without scotoma and from patients with MD. Stars provide significant differences in the post-hoc paired t-tests after a Bonferroni correction for multiple comparisons (see details in the text). *: $p < 0.05$, **: $p < 0.01$.*

Given the results reported in the two previous subsections of ‘Results’, it is not surprising that the ANOVA (within-subject factor: optic flow patterns (translational, rotational or radial), between-subject factor: group (patient or control)) for the motion coherence thresholds in patients and in controls without a simulated scotoma led to effects which are very similar to those reported for the comparison between patients and controls with a simulated scotoma. A highly significant effect of the pattern ($F(2,44) = 12.63$, $p < 0.001$) was observed, as well as a significant interaction between groups and optic flow patterns ($F(2,44) = 3.28$, $p = 0.029$). The group effect remained not significant ($F(1,22) = 2.32$, $p = 0.142$). Again, the pattern effect was mostly related to higher thresholds for translations. Post-hoc t-tests were largely in agreement with those reported for the comparison between patients and controls with a simulated scotoma. Patients notably had comparable thresholds than for controls for translational, rotational and radial patterns ($p > 0.05$).

Here as well, we controlled that extreme data points did not influence our results by reproducing our statistical analyses without these points. This operation left our conclusions unchanged.

Correlations between motion coherence thresholds estimated in patients with MD and clinical data

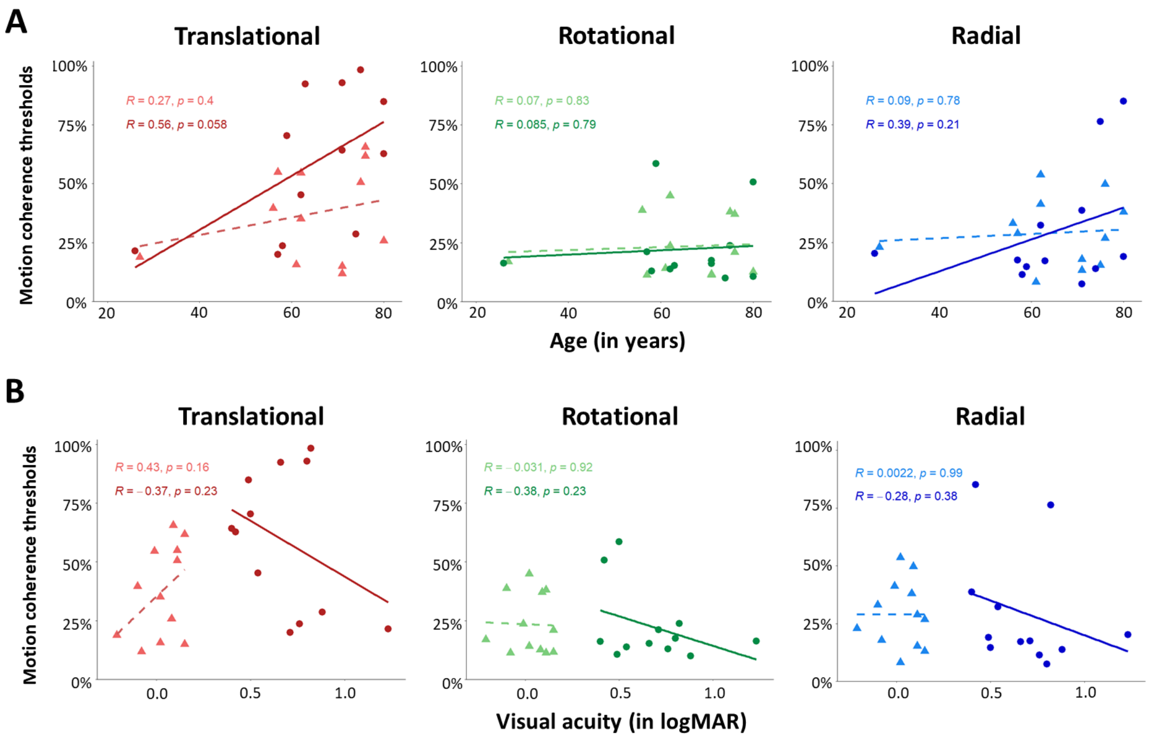

Figure S3. *A) Correlations between motion coherence thresholds and age (in years), estimated for translational (in red, leftward panel), rotational (in green, middle panels) and radial (in blue, rightward panels) patterns. Data points and associated regression lines are provided for patients (light colors) and also for their age-matched controls (dark colors). Pearson correlation coefficients (R) and associated p-values are provided on the upper parts of each panel. B) Correlations between motion coherence thresholds and visual acuity (in logMAR).*

Bias analysis

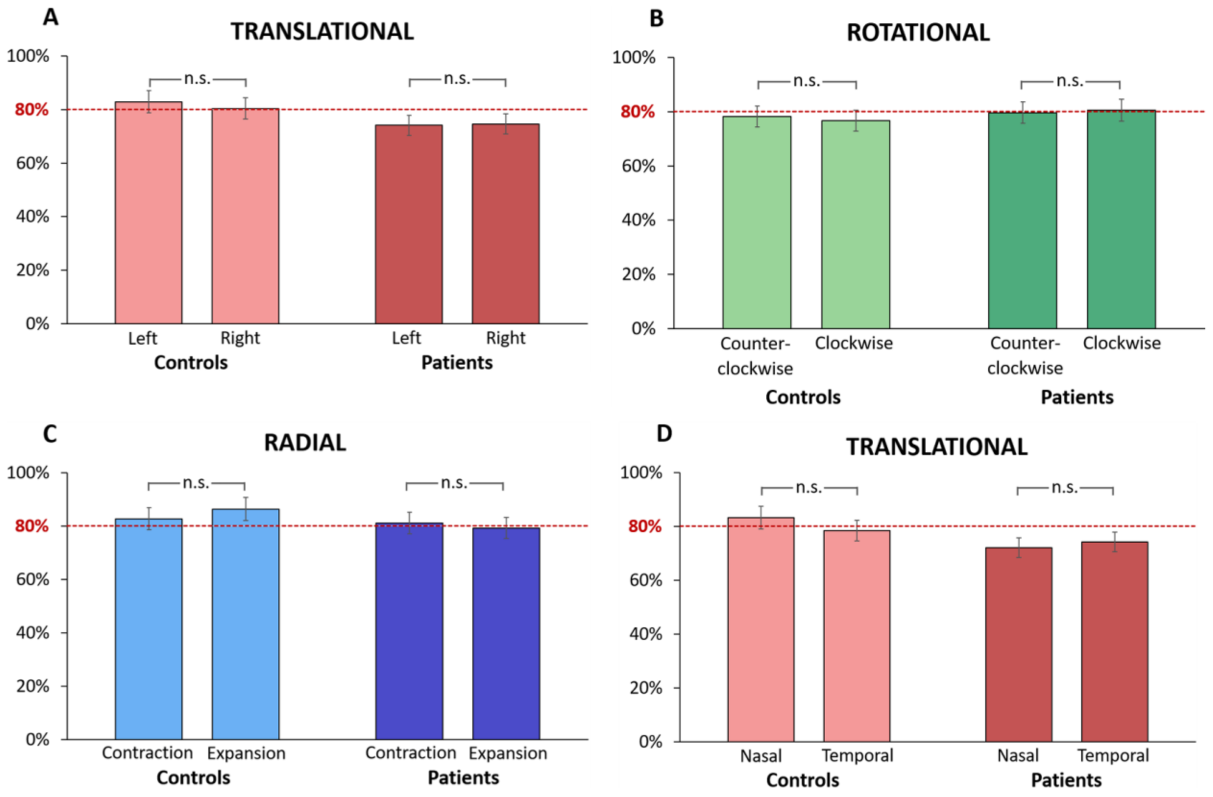

Figure S4. *Proportion of correct responses for (A) leftward and rightward motions (translational pattern), (B) clockwise and counterclockwise motions (rotational pattern), (C) contraction and expansion (radial pattern) and (D) motion along the nasal and temporal directions (translational motion). Light and dark boxes respectively represent data from age-matched controls (for stimuli with a simulated scotoma) and from patients with MD. Red lines correspond to a performance level of 80%. Black segments correspond to 95 percent confidence intervals on the distributions. Paired t-tests between the different data in controls and patients did not lead to any significant effects (n.s.: not significant).*

Motion coherence thresholds in men and women

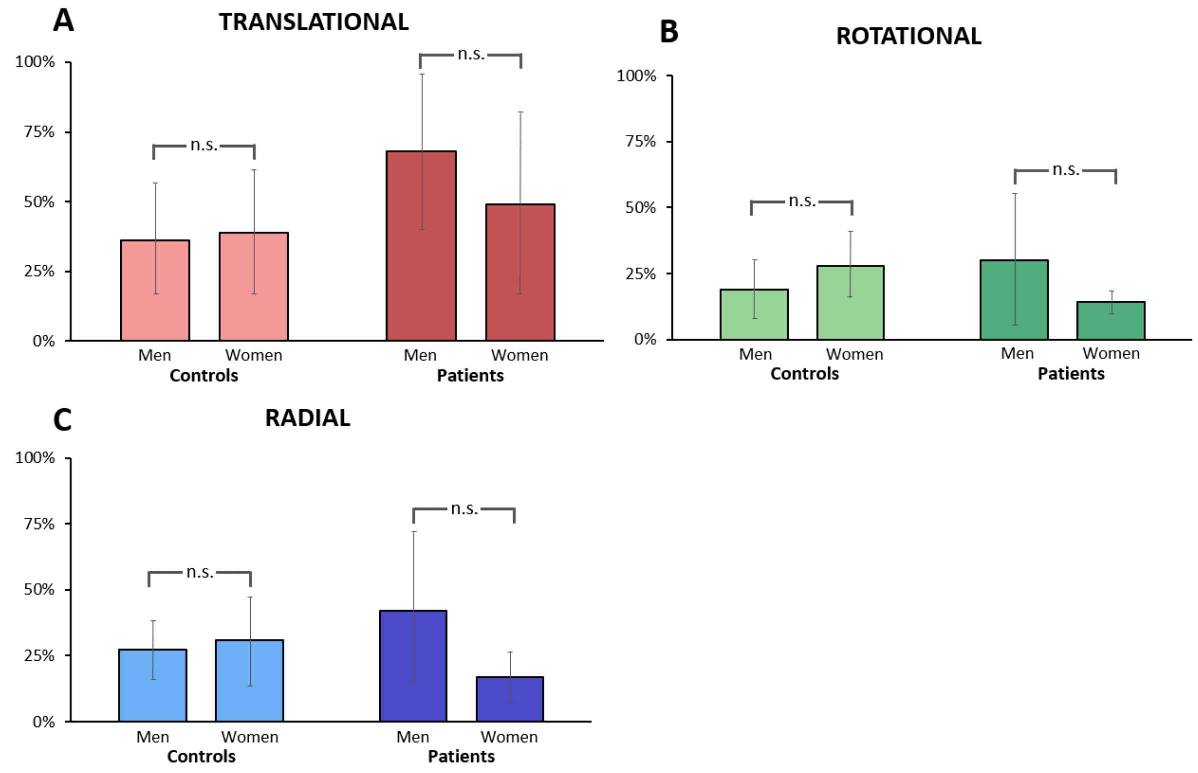

Figure S5. *Mean motion coherence thresholds in men and women for translational (A), rotational (B) and radial (C) patterns. Light and dark boxes respectively represent data from age-matched controls (for stimuli with a simulated scotoma) and from patients with MD. Black segments correspond to 95 percent confidence intervals on the distributions. Paired t-tests between the different data in controls and patients did not lead to any significant effects (n.s.: not significant).*

BIBLIOGRAPHY

46. Drasdo N, Fowler CW. Non-linear projection of the retinal image in a wide-angle schematic eye. *Br J Ophthalmol.* 1974:58(8),709-14.
